# Disaggregating asthma: Big investigation versus big data

**DOI:** 10.1016/j.jaci.2016.11.003

**Published:** 2017-02

**Authors:** Danielle Belgrave, John Henderson, Angela Simpson, Iain Buchan, Christopher Bishop, Adnan Custovic

**Affiliations:** a Department of Paediatrics, Imperial College, London, United Kingdom; b School of Social and Community Medicine, Faculty of Health Sciences, University of Bristol, Bristol, United Kingdom; c Division of Infection, Immunity and Respiratory Medicine, Faculty of Biology, Medicine and Health, University of Manchester, Manchester, United Kingdom; d Health Informatics, Faculty of Biology, Medicine and Health, University of Manchester, Manchester, United Kingdom; e Microsoft Research, Cambridge, United Kingdom

**Keywords:** Asthma, endotypes, machine learning, big data, birth cohorts, STELAR, Study Team for Early Life Asthma Research

## Abstract

We are facing a major challenge in bridging the gap between identifying subtypes of asthma to understand causal mechanisms and translating this knowledge into personalized prevention and management strategies. In recent years, “big data” has been sold as a panacea for generating hypotheses and driving new frontiers of health care; the idea that the data must and will speak for themselves is fast becoming a new dogma. One of the dangers of ready accessibility of health care data and computational tools for data analysis is that the process of data mining can become uncoupled from the scientific process of clinical interpretation, understanding the provenance of the data, and external validation. Although advances in computational methods can be valuable for using unexpected structure in data to generate hypotheses, there remains a need for testing hypotheses and interpreting results with scientific rigor. We argue for combining data- and hypothesis-driven methods in a careful synergy, and the importance of carefully characterized birth and patient cohorts with genetic, phenotypic, biological, and molecular data in this process cannot be overemphasized. The main challenge on the road ahead is to harness bigger health care data in ways that produce meaningful clinical interpretation and to translate this into better diagnoses and properly personalized prevention and treatment plans. There is a pressing need for cross-disciplinary research with an integrative approach to data science, whereby basic scientists, clinicians, data analysts, and epidemiologists work together to understand the heterogeneity of asthma.

A major obstacle to realizing precision (stratified or personalized) medicine in asthmatic patients is the lack of consensus in defining the disease, which is, at least in part, a consequence of “asthma” being an aggregated diagnosis comprising several different diseases.^1^, ^2^, ^3^, ^4^ It is now well established that both asthma^3^, ^5^, ^6^, ^7^, ^8^ and allergic sensitization^9^, ^10^, ^11^, ^12^ are umbrella terms (or syndromes) incorporating a variety of underlying endotypes sharing common symptoms and phenotypic characteristics.^13^, ^14^ Although by definition each endotype has unique pathophysiology and hence genetic and environmental associations,^13^, ^14^ it is likely that some mechanisms overlap 1 or more endotypes.^15^ This underlying heterogeneity is also reflected in responses to treatment. For example, a therapeutic agent might be specific for a pathway that is primarily responsible for the patient's asthma subtype, and therapeutic response can be predicted reasonably well by using relevant biomarkers,^16^, ^17^ such as the number of eosinophils in peripheral blood or sputum for mepolizumab^18^ or periostin levels for lebrikizumab.^19^ Alternatively, a therapeutic agent might be relatively nonspecific and target broad mechanisms shared between different asthma endotypes, in which case patients across different endotypes might display a spectrum of responses, which is likely the case with inhaled corticosteroids.

Across different disease areas, a vast number of genetic studies have initially raised expectations over “significant hits” that later delivered neither meaningful clinical diagnostic tools nor useful insights into disease pathogenesis.^20^ Genetic studies have thus far explained little of the heritability of complex diseases.^21^ Associated genetic variants generally have small effect sizes, and for many of these genetic variants, there is a lack of clear functional implication. In addition to gene-environment interactions,^22^ gene-environment correlations,^23^ and epigenetic mechanisms,^24^ the use of aggregated definitions of disease can also contribute to inconsistent findings between studies investigating genetic components of asthma. However, by using more specific phenotyping, a recent genome-wide association study identified an association of a specific asthma subtype characterized by early-life onset and recurrent severe exacerbations at preschool age, with a functional variant in the novel susceptibility gene *CDHR3* (rs6967330, C529Y).^25^ This genetic variant was associated with a greater risk of asthma hospitalizations in 2 birth cohorts, but there was no association with an aggregated definition of “doctor-diagnosed asthma.” Subsequent studies have shown that expression of human *CDHR3* facilitates rhinovirus C binding and replication and that a coding single nucleotide polymorphism in *CDHR3*, which was linked with asthma hospitalizations in birth cohort studies, mediates enhanced rhinovirus C binding and increased progeny yields *in vitro*.^26^ It is also of note that when asthma was disaggregated into subtypes, much stronger associations were observed for some of the genetic variants previously identified in genome-wide association studies, such as those in the 17q21 locus.^25^ The value of focusing on specific subgroups has been demonstrated in a study that showed that variants at 17q21 were associated with asthma but only in children who had rhinovirus-induced wheezing illness.^27^ Similarly, the risk of transient early wheeze, but not persistent wheeze, increases with the number of chronic obstructive pulmonary disease–associated alleles.^28^ Most of the genetic studies that used more precise phenotypes showed higher relative risk estimates than the modest effect sizes of genetic hits that were identified by using a simple binary trait definition of asthma, highlighting the need for a more refined subtyping of asthma to accurately identify genetic variants of clinical importance.^29^

Many environmental exposures are implicated in the development of asthma and in determining its severity.^30^, ^31^ As with genetic associations, there have been many inconsistent reports about the role of environmental exposures in asthmatic patients. We and others have shown that different phenotypes of childhood wheezing have different environmental associations.^2^, ^8^, ^32^, ^33^, ^34^, ^35^, ^36^, ^37^, ^38^ Similarly, different subtypes of atopic sensitization differ in their environmental risk factors; for example, endotoxin exposure is protective for multiple early but not multiple late sensitizations.^39^ It is likely that the effect of most environmental factors varies across subjects with different genetic predispositions, but the precise nature of most gene-environment interactions remains unclear.^22^ One of the most replicated findings of gene-environment interactions in the development of allergic sensitization is between *CD14* variants and environmental endotoxin exposure.^40^ Several studies have reported that high endotoxin exposure can protect against sensitization but only among subjects with a specific genetic predisposition (C allele homozygotes of rs2569190).^40^, ^41^ However, in the same genotype group the effect of endotoxin exposure differed by phenotype, decreasing the risk of atopic sensitization and eczema but increasing the risk of nonatopic (but not atopic) wheezing.^41^ Other examples that the nature of gene-environment interactions can differ between different wheeze phenotypes include the finding that day care attendance can have opposite effects on atopic wheezing among subjects with different genetic variants in the Toll-like receptor 2 gene (being protective in some but increasing the risk in others),^42^ with no such effect being observed for nonatopic wheezing.^42^ This suggests that replication of gene-environment interactions can be improved through a more precise definition of the outcome of interest.^43^ The lessons for intervention studies aimed at personalized prevention is that individual genetic predisposition must be taken into account when seeking the environmental protective/susceptibility factors amenable to intervention^30^ and that interventions that might be effective in one subtype of wheezing might not necessarily work for other subtypes.

One area that has been relatively more successful is the identification of biomarkers^16^ for more targeted treatment strategies.^17^ A recent review Berry and Busse^44^ identified 4 main biomarkers that might help optimize treatment strategies for different asthma phenotypes. These biomarkers are generally limited to T2 mechanisms: eosinophils, exhaled nitric oxide, periostin, and IgE. However, biomarker assessment has not as yet become an integral part of clinical practice, nor is it reflected in current asthma guidelines. Validation steps are necessary, and acknowledgement in asthma guidelines would prompt application of such information in clinical practice. The identification of non-T2 biomarkers is an important area of research that needs to be exploited^44^ with biomarker identification for asthma and allergic diseases still in its embryonic stages. Furthermore, although biomarker identification has indeed led to more targeted asthma treatment strategies, there are currently no biomarkers that reflect the underlying causal mechanisms, which could predict disease onset or progression.

Although phenotypic heterogeneity of asthma is now widely accepted, we are still scratching the surface of identifying the different endotypes of asthma and understanding their unique underlying pathophysiologic mechanisms, which is a prerequisite for precision medicine.^15^ Although there is general consensus that there are different asthma endotypes and different phenotypes of wheezing during childhood, there is no consensus on how best to define them. A more refined endotypic definition of asthma and allergic diseases can drive more targeted research to identify distinct molecular, genetic, environmental, and demographic characteristics that might allow us to predict causality of distinct endotypes with greater accuracy.^45^

One approach used in a number of studies has been to investigate temporal patterns of symptoms over time. The common labels across most studies have been transient early wheeze, late-onset wheeze, and persistent wheeze.^46^ However, different studies reported different numbers of childhood wheeze phenotypes (eg, ranging between 2 and 6).^2^, ^46^, ^47^ One of the challenges in current research aimed at defining subgroups of patients based on the natural history of wheezing is the lack of consistency in definition of these phenotypes and what they represent. The inconsistency in defining wheeze phenotypes based on longitudinal profiles of symptoms over time across different studies might merely reflect inconsistencies in the nature and timing of questions used (eg, physician-confirmed wheezing^8^, ^34^ vs parentally reported wheezing^6^, ^36^). Thus although the definition of subtypes based on profiles of symptoms over time is better than that based on a single time point, variability in input variables has an effect on the accuracy of defining subtypes and identifying predictive models.^2^, ^47^, ^48^, ^49^

## Can “big data” provide solutions?

Big data refers not only to the ready availability of large volumes of routine health care data being rapidly generated but also to the complexity of these data, which is evident in the amplified scale of biological, genetic, environmental, and phenotypic data. The scale of these data often makes handling, management, and analysis challenging with the use of standard statistical methods. The evolution of powerful computational tools to analyze such high-dimensional large data sets has pushed the boundaries of endotype discovery. Such data provide the potential for “learning” patterns or predicting health outcomes and optimal treatment strategies based on prior information. However, one of the major challenges of big data remains the bias inherent to its volume. Furthermore, the vast increase in the quantity of data generated has made it impossible at times to know for what we are looking and what questions need to be asked. As a consequence, data-driven hypothesis-generating approaches to understanding disease are overshadowing traditional hypothesis-based research (hypothesis testing) through carefully constructed questions and observations. In a hypothesis-generating approach to data analysis, we look for structure in the data without necessarily having a specific research hypothesis we want to verify. This is an advantage where, for example, we have measures of multiple biomarkers but are uncertain of the role of these biomarkers in predicting asthma. A hypothesis-generating approach can be used to identify patterns in biomarkers (eg, which ones are similar or which ones modify the effect of other biomarkers) to predict the disease. In recent years, big data has been sold as a panacea for generating hypotheses and driving new frontiers of health care; the idea that the data must and will speak for themselves is fast becoming a new dogma. However, we argue for combining data-driven and hypothesis-driven methods in careful synergy.

## On methodologies: Understanding reality versus predicting the future

Machine learning, computational statistics, biostatistics, a traditional approach to epidemiology, and clinical and biological expertise can elucidate different aspects of the same problem. Machine learning is a data-driven approach to identify structure within data to make predictions and identify patterns. It is used commonly by computer scientists for problem solving in a variety of fields and is used increasingly to disaggregate complex disease phenotypes in respiratory medicine and allergy.^1^, ^3^, ^5^, ^10^, ^11^, ^12^ It must be noted that although machine learning as a discipline is fairly new, the mathematic and statistical foundations have been in existence since the beginning of the 20th century.^50^, ^51^, ^52^, ^53^ Machine learning as a new discipline is a result of the exponential growth in computational power, which has enabled implementation of the mathematic groundwork that was initiated decades earlier.^54^, ^55^

One of the (somewhat artificial) distinctions between machine learning and conventional statistical approaches is that although machine learning focuses on prediction models and attempts to accurately predict future outcomes/events (whether these be future disease states or the development of symptoms in later life), statistics tends to focus on extant observations and constructing models to aid understanding of the data and the current status of disease. Hence statistics tends to focus on causality and associations in an attempt to explain the disease and to understand uncertainty in the modelling assumptions.

Modelling assumptions refers to our framework for representing the data or research questions related to that data. Because these are assumptions, we are uncertain about them and need some way of testing whether these assumptions are true. Machine learning applied to medicine attempts to predict disease states and to get the best estimate of uncertainty analogous to clinical diagnosis. Both approaches combined with epidemiology, which carefully tests hypotheses to infer causality, need to be considered along with medical and biological expertise in a holistic understanding of disease.

## Bayesian versus frequentist approach to understanding disease etiology

We now introduce the reader to 2 different approaches to hypothesis testing and hypothesis generation: the Bayesian and frequentist approaches. The aim of this discussion is to provide a conceptual framework that is currently commonly used in statistical and machine learning and can be applied to both big and small data sets in health care research. An understanding of these 2 paradigms is formative for a team approach to understanding disease etiology in health care.

The frequentist paradigm is an unconditional perspective, meaning that it assumes that the observed data are representative of the population with an independent and identical distribution. Thus this paradigm, as the name suggests, emphasizes the frequency of the data. On the other hand, the Bayesian approach uses probability as a principled framework for quantifying our uncertainty of the data and of the true estimated effects in our models, thereby allowing the explicit incorporation of prior scientific knowledge into statistical reasoning. Bayesian models provide a framework whereby prior knowledge and data from previous studies can be incorporated explicitly with the data at hand in the analytic model to formulate a posterior distribution that takes account of both the observed data and prior knowledge.^56^ The inherent characteristics of the Bayesian approach to data analysis make this framework more amenable to handling large-scale problems and easily extending the complexity of current models that use classical statistical or frequentist tools. Although the frequentist approach also relies on prior clinical knowledge, the difference is that this approach does not seek to explicitly quantify this knowledge; it only relies on the data at hand for incorporating assumptions we make about the statistical models for the data.

In understanding the etiology of asthma and allergic disease, Bayesian models provide a flexible and unified framework for understanding the probability of disease manifestation and comanifestation, incorporating evidence from the literature or hypotheses from medical experts^5^ through the explicit quantification of this evidence. However, although Bayesian methods provide an intuitive and unified platform for carrying out statistical research, the results are often computationally intractable and resolving these is active area of research.^57^, ^58^, ^59^, ^60^ The exponential increase in computational power and the increasing availability of tools that can handle large-scale data has facilitated the use of Bayesian methods, and it is important that we capitalize on these relatively complex tools to improve human health.

The use of Bayesian statistics is relatively uncommon in the medical literature, in part because of the greater complexity involved in using these models. One of the strengths of the Bayesian approach is that it can be used to enrich our current understanding of disease, with its capacity to elicit robust scientific inference by encouraging the user to think about the underlying statistical and scientific problems^61^ by assigning explicit quantities to scientific assumptions. This might provide a powerful tool for extending model complexity to reflect the underlying complexity of the data and the scientific problem being addressed. Bayesian methods allow the clinician to take an active role in the modelling process by quantifying prior probabilities based on expert assessments. However, one limitation of Bayesian analysis is the difficulty in eliciting this prior knowledge and quantifying expert knowledge,^62^, ^63^ mainly because pf the training and time necessary to develop “informative” prior knowledge. In some cases this can be more expensive than collecting more data. This approach is not unique to Bayesian analysis. Prior knowledge can be integrated to a less explicit degree by using a frequentist platform, where, rather than specifying or quantifying expected results, a clinician/topic expert could specify explicit assumptions about expected transitions of allergic disease and symptom profiles, as well as the proportions of patients with different profiles and severities of disease. In this sense the often-acclaimed advantage of Bayesian analysis as being able to incorporate informative prior knowledge based on expert knowledge may be overstated. The frequentist approach can be used to compare different model assumptions based on expert knowledge, which might be a more pragmatic approach than trying to quantify uncertainty surrounding the size of an effect. The important take-home message is that in weighing up the Bayesian and frequentist approach to statistical modelling, the question is not so much which method is best but rather which method is more appropriate for the question being addressed and for encapsulating model complexity with parsimony.^64^

## Away from methodological polemics toward data science

This dissonance between machine learning, biostatistics, and epidemiology on the one hand and between the Bayesian and frequentist paradigms on the other presents artificial dichotomies. Beyond the methodological dogma, science needs to be pragmatic, selecting the right method or methods for the problem/question. Different methodologies are not mutually exclusive; indeed, an ensemble of methods might be more effective for identifying distinct subtypes of diseases. Data science must take the path of least inferential resistance, including the use of better ways to incorporate prior knowledge about likely causal mechanisms.

## Latent variable modelling approach to understanding subtypes of disease

A general area in which Bayesian and frequentist paradigms compete (or complement) is latent variable modelling.^65^ This section highlights the importance of latent variable modelling as a generalized framework for hypothesis generating and dimensionality reduction. Dimensionality reduction is an important tool for analysis of big data, in which we have multiple clinical, molecular, genetic, environmental, and phenotypic elements (ie, high dimensions). As the name suggests, in dimensionality reduction the aim is to reduce the dimension of the data set to a more manageable group of meaningful variables. Latent variable modelling can also be used not just to reduce the dimension of variables within a large data set but also to identify subgroups of patients based on patterns within these variables. Latent variable models are increasingly cited in the medical literature^66^, ^67^, ^68^ for classifying different phenotypes and subphenotypes of diseases based on individual disease profiles. The latent variable model is a statistical model in which the observed association between (manifest) observed variables is regarded as spurious because this observed association can be explained by an indirectly observed, hidden, or latent variable rather than being causally related. This provides a powerful approach to probabilistic modelling and offers a flexible method to investigate substructures within complex data sets, in which associations between a set of observed variables are supplemented with additional latent variables. Therefore latent variable modelling allows us to move from hypothesis testing to hypothesis generation.^69^ A further advantage of using latent variable modelling is that it is easier to represent high-dimensional parameters^70^, ^71^, ^72^, ^73^ on a reduced space with fewer dimensions. For example, using such techniques, we can reduce the dimensionality of multiple continuous variables into a more manageable set with fewer variables (parameters). The reduced number of variables is representative of a larger data set. Reducing dimensionality onto a latent space in turn facilitates the interpretation of multiple correlated continuous factors. The use of Bayesian methods in this context complements the likelihoods from the data with prior hypotheses about the expected distribution of these latent variables. We have successfully used generalized latent variable modelling approaches to identify distinct subtypes of asthma^2^, ^3^, ^6^, ^9^, ^10^, ^11^, ^12^, ^15^, ^34^, ^36^ and allergic diseases.^5^, ^9^, ^11^, ^12^ The key to future discoveries is to uncover underlying pathophysiologic mechanisms (endotypes) that drive these distinct subtypes.^1^

## Big data with big promises: The contribution of cohorts to our understanding of asthma

The public's expectation that their health data should be used to improve care services has sometimes been stalled by fears over privacy and unregulated commercial uses of the data.^74^ Birth cohort studies are an interesting parallel because cradle-to-grave health care records can be thought of in this way. However, unlike routine health care records, birth cohorts make more systematic observations before the onset of disease, facilitating exploration of the natural history of disease. With data from birth cohorts, investigators can follow development of disease over time, which mimics clinicians' diagnoses and follow-up observations but in a more anticipatory way.

**Fig 1:**
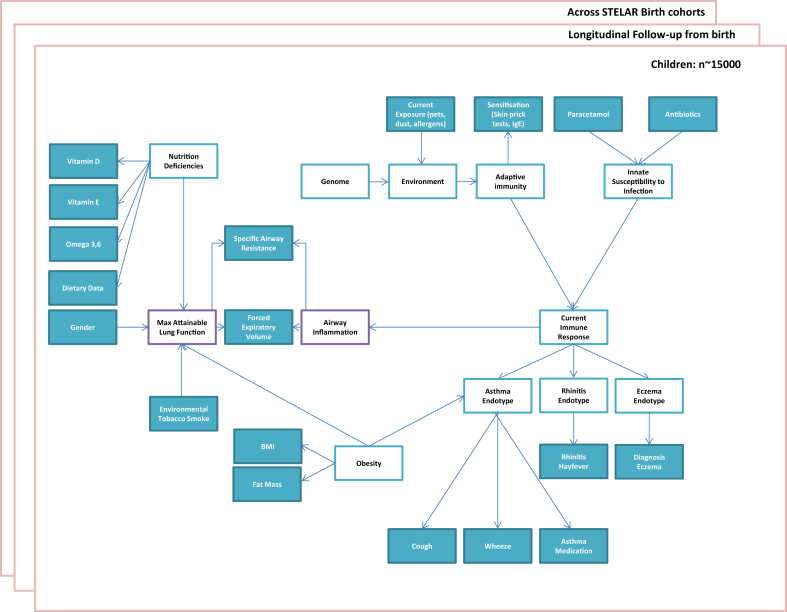
Roadmap of challenges to understanding asthma and allergic diseases within the STELAR consortium.

One initiative aimed at harnessing data from birth cohorts to understand the development of different endotypes of asthma and allergic diseases is the Study Team for Early Life Asthma Research (STELAR) consortium.^15^ STELAR combines data from 5 United Kingdom birth cohorts aimed at understanding the development of asthma and allergic diseases through the life course. The cohorts include the Avon Longitudinal Study of Parents and Children, Ashford cohort, Isle of Wight cohort, Manchester Asthma and Allergy Study, and Aberdeen Study of Eczema and Asthma to Observe the Effects of Nutrition. STELAR has data on more than 14,000 participants with repeated measures on symptoms of asthma and allergy over multiple time points from childhood into adulthood. An important feature is that participants are sampled from the general population, enabling generalizable conclusions about the pathophysiology and development of asthma at large. This would be difficult with routine health care records because they have more selected/biased observations sampled later in the natural history of disease. Fig 1 summarizes the challenges in understanding asthma and allergic disease that will drive future research in the STELAR consortium.

An important area in which recent cohort studies have elucidated pathways for development of asthma into fixed airway obstruction is in investigating longitudinal profiles of lung function.^75^, ^76^, ^77^ Such profiles can shed light on the causes and consequences of airway obstruction that provide us with an objective marker of airway disease, which can be easily translated into clinical practice.

We consider that clinical/case (patient) cohorts and birth cohorts provide complementary windows on different aspects of understanding disease etiology.^78^ An important and largely unanswered question is how best to translate findings between case and birth cohorts (ie, between clinical and general populations) to inform better prevention and early intervention strategies.^79^ The case has been argued for automated methods to update disease models in real time.^80^, ^81^, ^82^, ^83^, ^84^ The technologies are available, but they have not been applied in this way to accelerate the translation of research findings into clinical practice nor have they been used to enrich research models with emergent clinical phenomena. The importance of carefully characterized birth and patient cohorts with genetic, phenotypic, biological, environmental, and molecular data cannot be overemphasized in the quest to understand asthma and discover its endotypes.

## Conclusion: The importance of team science

**Fig 2:**
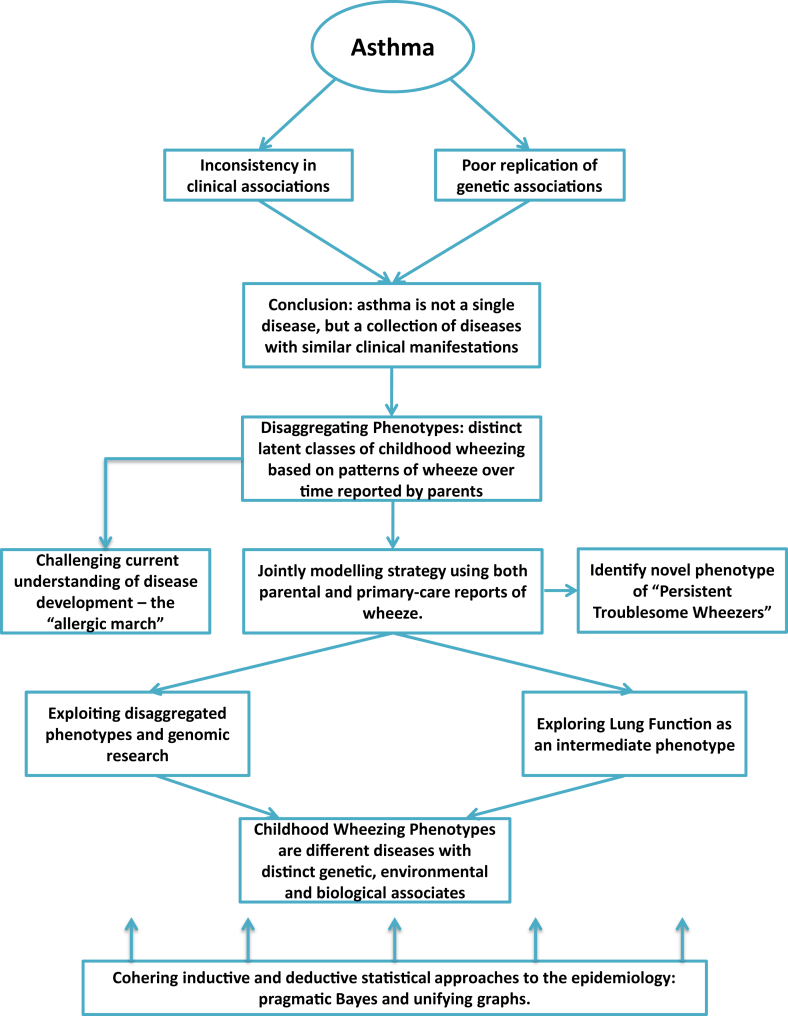
Data cycle: an integrative approach to understanding disease endotypes.

We are facing a major challenge to bridge the gap between identifying subtypes of asthma in clinical and general populations (and to find ways to translate the findings between these 2 contexts) to understand causal mechanisms of the discovered subtypes and translating this knowledge into better prevention and management strategies.^78^, ^85^ To this effect, understanding disease causality within the data analytic framework is fundamental to improve our understanding of asthma endotypes and their distinct etiologies.^86^ From this perspective, significant investment needs to be made in advancing statistical and computational tools to solve health care problems. However, although advances in computational methods can be valuable for identifying unexpected structure in data to generate hypotheses, there remains a need for interpreting results with scientific rigor and testing hypotheses that arise from this process. One of the dangers of ready accessibility of health care data and computational tools for analyzing these data is that the process of data mining can become uncoupled from the scientific process of clinical interpretation, understanding the provenance of the data, and external validation.^87^ There is a pressing need for cross-disciplinary research to avoid the false idol of big data being the single source of truth. A more credible approach is to blend big data with big reasoning, so that prior structure is imposed on the data meaningfully. Fig 2 illustrates a data cycle encompassing the problem: an integrative approach to data science whereby basic scientists, clinicians, biostatisticians, and epidemiologists work together to understand the heterogeneity of asthma and allergic disease. Given that big data takes team science, it would be important for the scientific and academic communities to reassess systems and criteria for promotion that still, in many cases, do not give sufficient credit for perceived nonleadership roles.

We need to ensure that we harness bigger health care data in ways that produce meaningful clinical interpretation and to translate the findings into better diagnoses, biomarkers, and properly personalized prevention and treatment plans. As an example, big data could be used to identify patients with exacerbations and inadequately controlled asthma and then prompt evaluation of their treatment regimen.^17^ One of the advantages of big data is its capacity to change the way we currently do clinical research in asthma through building more robust predictive models to understand subtypes of this complex disease. The direction needs to move away from looking at average effects (which is a strategy commonly used in randomized clinical trials that make use of stringent exclusion criteria as their modelling framework). We advocate that research into causal biomarker identification and optimal management and prevention strategies needs to be anchored in understanding of the underlying disease heterogeneity.

It is important that we, as a community of health care professionals, work toward transferring evidence-based information to better patient care. Therefore clinical practitioners should be aware of the need to treat asthma and other heterogeneous diseases in a more personalized manner and be ready to incorporate the discovered stratified medicine strategies in a timely fashion.
